# Time-Order Errors in Duration Judgment Are Independent of Spatial Positioning

**DOI:** 10.3389/fpsyg.2017.00340

**Published:** 2017-03-09

**Authors:** Charlotte Harrison, Nicola Binetti, Isabelle Mareschal, Alan Johnston

**Affiliations:** ^1^Department of Experiment Psychology, University College LondonLondon, UK; ^2^School of Biological and Chemical Sciences, Psychology, Queen Mary University of LondonLondon, UK; ^3^School of Psychology, University of NottinghamNottingham, UK

**Keywords:** visual perception, time perception, visual short-term memory (VSTM), retinotopy, spatiotopy, time-order errors

## Abstract

Time-order errors (TOEs) occur when the discriminability between two stimuli are affected by the order in which they are presented. While TOEs have been studied since the 1860s, it is unknown whether the spatial properties of a stimulus will affect this temporal phenomenon. In this experiment, we asked whether perceived duration, or duration discrimination, might be influenced by whether two intervals in a standard two-interval method of constants paradigm were spatially overlapping in visual short-term memory. Two circular sinusoidal gratings (one standard and the other a comparison) were shown sequentially and participants judged which of the two was presented for a longer duration. The test stimuli were either spatially overlapping (in different spatial frames) or separate. Stimulus order was randomized between trials. The standard stimulus lasted 600 ms, and the test stimulus had one of seven possible values (between 300 and 900 ms). There were no overall significant differences observed between spatially overlapping and separate stimuli. However, in trials where the standard stimulus was presented second, TOEs were greater, and participants were significantly less sensitive to differences in duration. TOEs were also greater in conditions involving a saccade. This suggests there is an intrinsic memory component to two interval tasks in that the information from the first interval has to be stored; this is more demanding when the standard is presented in the second interval. Overall, this study suggests that while temporal information may be encoded in some spatial form, it is not dependent on visual short-term memory.

## Introduction

Time-order errors (TOEs) of apparent duration occur when two successive stimuli are compared (Jamieson and Petrusic, 1975). In a typical TOE experiment, a standard and a comparison stimulus are displayed sequentially, separated by an inter-stimulus interval (ISI). Participants are then asked to judge if the comparison was, for example, louder or softer than the standard (Hellström, 1985). First described by Fechner in the 1860s, early findings showed evidence of TOEs in many different domains, including estimations of length, loudness, brightness, and duration (Needham, 1934; Woodworth and Schlosberg, 1938). Generally, results showed that with brief intervals the first interval is overestimated and/or the second is underestimated, with the reverse being true for longer intervals (Allan, 1979). The size of the TOE decreases as the ISI increases, and trial feedback causes them to not be observed at all (Allan and Gibbon, 1994).

Several explanations for TOEs have been suggested. The first proposes that errors occur due to personal biases, such as an overall bias in answering. For example, maybe people are more prone to saying a duration lasted longer than they are to saying that it was shorter (Allan and Gibbon, 1994). While there had been some support for this in the literature (e.g., Masin et al., 1988), it has been disputed by Jamieson and Petrusic (1975), who demonstrated this could not (at least wholly) be the case, as TOEs are consistent even when instructions are changed to undermine such biases. They suggest that it is perceptual effects that make a second stimulus appear proportionally different to the first, and that TOEs are more readily explained by the fading or assimilation of a memory trace of the stimulus that was initially presented. This has been supported by other studies (Ornstein, 1969; Schab and Crowder, 1988). Explanations based on memory traces are further supported by findings related to ‘virtual standards,’ which also showed that duration discrimination thresholds are greater when a comparison stimulus is presented before the standard, compared to the reverse (Nachmias, 2006).

Temporal and spatial processing have typically been treated as separate in traditional models of time perception (Creelman, 1962; Treisman, 1963). However, more recently evidence has grown in favor of temporal models that point to the existence of separate domain-specific timing mechanisms (Meck, 2005). For example, it has been shown that apparent duration of brief intervals can be manipulated in spatially specific regions of visual space (Johnston et al., 2006), indicating that temporal processing does involve some sort of spatial component. Initial findings demonstrated that this occurs in a retinotopic frame of reference (Ayhan et al., 2009), though it has been suggested that adaptation-based apparent time compression may also occur in a spatiotopic frame (Burr et al., 2007; Latimer and Curran, 2016).

If, as evidence suggests, TOEs stem from degradation of memory traces, spatial positioning of briefly presented stimuli may influence them, as they will be subject to the effects of working memory. Being able to use visual inputs to guide behavior requires working memory to act as a temporary buffer that can hold sustained information from across saccades as well as other visual interruptions (Xu and Chun, 2006). This is essential to be able to perceive a stable world around us despite drastically changing retinal feedback (Golomb et al., 2008). While visuospatial memory has a high capacity for storing sensory information, its ability to hold features in short-term working memory is limited (Phillips, 1974; Alvarez and Cavanagh, 2004).

Both retinotopic and spatiotopic buffers have been suggested to exist within visual short-term memory (Feldman, 1985). It is envisaged that the retinotopic buffer stores coordinates from the original information, and then at the time of a saccade the spatiotopic buffer becomes activated, helping to create a steady representation of the world (McRae et al., 1987). The idea of an explicit spatiotopic memory has been proposed several times (Breitmeyer et al., 1982; Melcher, 2007), and receives support from the finding that in infancy there is a bias toward retinotopic representations that precede the development of higher-order spatiotopic and body centered representations (Kaufman et al., 2006). However, trying to assess the relative impact of each system is confounded by the fact that the two are not dissociable until an eye movement has been made (Golomb et al., 2008). When looking at how visual spatial information is processed and retained, more recent studies have suggested that memory is significantly more accurate and precise in retinotopic, compared to spatiotopic, coordinates and that spatiotopic but not retinotopic error accumulates with each eye movement (Golomb and Kanwisher, 2010).

Given that TOEs may be driven by memory traces, and timing mechanisms have been shown to be spatially localized, we asked whether there would be any effect of spatial repetition on perceived duration; either by improving performance due to accessing the same mechanism twice or by degrading performance due to interference or overwriting of stored information. In the current experiment, participants were asked to judge which of two presented stimuli lasted longer. We investigated whether duration discrimination and the stimulus order error of the two intervals would be significantly different for stimuli placed at overlapping spatial positions – in both retinotopic or spatiotopic coordinates – compared to those that are placed at spatially separate locations. Further, we hypothesize a difference may be found between stimuli placed in retinotopic and spatiotopic regions, due to retinotopic memory being more accurate and precise.

## Materials and Methods

### Participants

Six participants, two male and four female, took part in the study (*M* = 26.1 years, range = 22–33 years). One participant (CH) took part in all conditions.

### Materials

Stimuli were presented on a Sony GDM-F520 CRT monitor driven by a ViSaGe MKII Stimulus Generator (Cambridge Research Systems) ^1^. The experimental software was written using MATLAB 2012b (MathWorks) ^2^ and the CRS toolbox. Responses were recorded using a wireless CT6 Response Box and infrared receiver (Cambridge Research Systems). Participants used a chin rest, positioned at a 57 cm distance from the screen.

### Stimuli and Design

**FIGURE 1:**
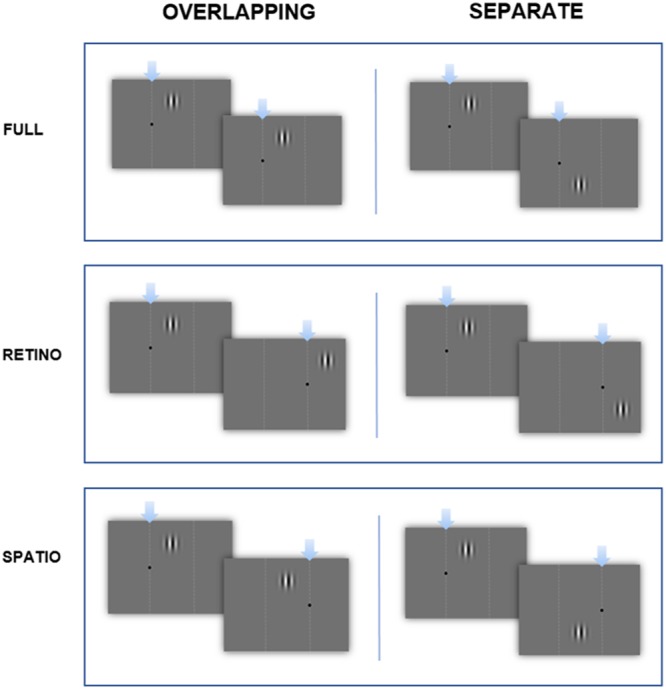
**Time-course of the experiment.** Examples of: ‘Full’ overlapping and separate conditions (fixation point doesn’t move, stimuli in same panel), ‘Retinotopic’ overlapping and separate conditions (fixation point moves, stimuli in same panel) ‘Spatiotopic’ overlapping and separate conditions (fixation point moves, stimuli in different panels). Dashed lines for illustration only.

Two circular sinusoidal gratings with a spatial frequency of one cycle/degree were presented sequentially on a gray screen, using a Gaussian filter for stimulus onset and offset. Each consecutive stimulus drifted in counter-phase to the other. Gratings could be presented on the screen in (1) retinotopic, (2) spatiotopic, or (3) ‘full’ (both retinotopic and spatiotopic) coordinates relative to a fixation point (see **Figure 1** for details). Each of these trial types could occur within an overlapping or separate spatial frame. The ‘separate’ conditions helped to control for amount of eye movement and horizontal displacement of the stimuli in the trials. Test stimuli were displayed for a variable duration between 300 and 900 ms, in intervals of 100 ms. This was compared with a standard stimulus of 600 ms. We derived a psychometric function from the data and extracted the point of subjective equality (PSE) as a measure of the apparent duration of the standard and the slope, which provided a measure of duration discrimination. Order of presentation (whether a standard or comparison stimulus was displayed first) was randomized on a trial-by-trial basis. Each level of the comparison stimuli was presented 30 times, giving a total of 210 trials per block. Different trial types were tested separately, giving 12 block-types in total.

### Procedure

Participants were each tested on 6 of the 12 possible blocks types. Block sequence was counterbalanced between participants. Each block lasted between 15 and 20 min. Participants were shown where the stimuli would appear in each block and allowed time to practice the task until they felt comfortable. On each trial after the two gratings appeared on the screen they were told to make a button press response indicating whether they thought the first or second stimulus they viewed had lasted longer. Testing took place over two 1-h sessions, with three testing blocks in each.

## Results

### Stimulus Order Effect

**FIGURE 2:**
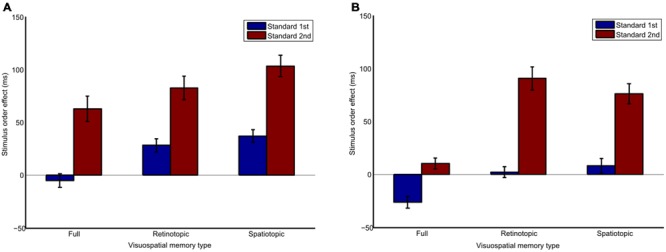
**Bar graph of differences in the stimulus order effect the full, retinotopic and spatiotopic-based conditions, in (A)** overlapping and **(B)** separate spatially placed trials when the standard stimulus is presented 1st or 2nd. Positive values indicate the second interval appears to be longer than the first. Error bars show std. error.

The stimulus order effect, expressed as the apparent duration of the second interval relative to the apparent duration of the first interval is plotted in **Figure 2**. A three-way repeated-measures ANOVA was conducted to test for differences in the magnitude of the stimulus order effect. There was a significant main effect due to differences between full (*M* = 10.52, *SD* = 15.65, 95% CI [-27.76, 48.81]), retinotopic (*M* = 51.04, *SD* = 25.28, 95% CI [-10.82, 112.91]), and spatiotopic (*M* = 61.18, *SD* = 19.47, 95% CI [13.53, 108.82]) visuospatial memory types, *F*(2,12) = 4.67, *p =* 0.032, $\eta_{p}^{2}$ = 0.438. Pairwise comparisons, with respect to the main effect of visuospatial memory type, revealed that the full condition was significantly different from both the retinotopic (*p* = 0.02) and spatiotopic (*p* = 0.022) conditions, indicating the stimulus order effect was smaller for the full condition compared to the other two. There was no significant difference of spatial frames between the overlapping (*M* = 26.93, *SD* = 13.32, 95% CI [-5.67, 59.53]) or separate (*M* = 54.9, *SD* = 27.26, 95% CI [-11.81, 121.61]) conditions, *F*(1,12) = 1.38, *p* = 0.285. Finally, there was a significant effect of stimulus order between standard 1st (*M* = 7.43, *SD* = 7.9, 95% CI [-12.06, 26.92]), and standard 2nd (*M* = 74.4, *SD* = 30.8, 95% CI [-0.97, 149.77]) conditions, *F*(1,6) = 5.98, *p =* 0.05, $\eta_{p}^{2}$ = 0.499, indicating the stimulus order effect was greater when the standard stimulus was presented second. No significant interactions were found.

### Duration Discrimination

**FIGURE 3:**
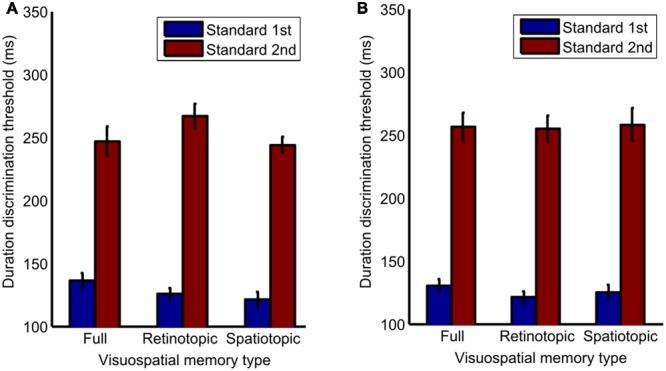
**Bar graph of differences in standard deviation between the full, retinotopic and spatiotopic conditions, in (A)** overlapping and **(B)** separately spatially placed trials when the standard stimulus is presented 1st or 2nd. Error bars show std. error.

Next, a three-way repeated-measures ANOVA was conducted to test for differences in duration discrimination thresholds. There was no significant main effect of visuospatial memory type between the full (*M =* 192.75, *SD* = 19.21, 95% CI [145.75, 239.75]), retinotopic (*M =* 192.87, *SD =* 14.37, 95% CI [157.71, 228.03]), and spatiotopic conditions (*M* = 187.65, *SD* = 22.06, 95% CI [133.66, 241.64]), *F*(2,12) = 0.053, *p* = 0.949. There was no significant effects between overlapping (*M =* 190.85, *SD* = 190.86, 95% CI [165.14, 216.58]) and separate spatial placement (*M =* 191.32, *SD* = 23.98, 95% CI [132.64, 250.01]), *F*(1,6) = 0.001, *p* = 0.982. However, there was a significant main effect of stimulus order between trials where the standard was presented first (*M* = 127.05, *SD* = 5.36, 95% CI [186.85, 323.4]) and where the standard was presented second (*M* = 255.13, *SD* = 27.9, 95% CI [113.94, 140.16]), *F*(2,12) = 25.41, *p* = 0.002, $\eta_{p}^{2}$ = 0.809. No significant interactions were found. See **Figure 3** for details.

## Discussion

The current study aimed to investigate whether TOEs can be affected by the spatial positioning of the stimuli being compared. We expected to find a difference in the magnitude of the stimulus order error, or in duration discrimination, on trials presented in an overlapping spatial frame compared to separate, due to the recruitment of visuospatial working memory. We further predicted that there would be a difference between the retinotopic, spatiotopic, and ‘full’ conditions. It was found that stimulus order effects were greater in the retinotopic and spatiotopic conditions compared to the full condition. One factor that differentiated these conditions is the absence of a saccade in the full condition, suggesting the occurrence of an eye movement between two intervals influences the magnitude of the TOE, creating a larger bias. This may reflect the known effect of chronostasis (Yarrow et al., 2001).

Significant differences in stimulus order error and duration discrimination thresholds were found for the order of presentation; TOEs were larger, and duration discrimination was diminished when the standard stimulus was presented second in a trial rather than first, no matter which frame of reference the stimuli were presented in. This is in line with previous findings have shown that the duration discrimination threshold is higher when a standard stimulus is presented in the first rather than the second interval. This occurs whether presentation order is blocked or randomized (Nachmias, 2006). These order effects are thought to occur due to participants referring to an implicit standard rather than a presented standard for their judgments. The results confirm that the presentation order of the stimulus determines the sensory precision of a temporal judgment. There is an intrinsic memory component to two interval tasks; the information from the first interval has to be stored, and therefore the task is more demanding when the standard is presented in the second interval.

However, no significant difference was found between overlapping and separate spatial frames. Previous research has found that visuospatial memory for information stored in retinotopic coordinates is more accurate and precise (Golomb and Kanwisher, 2012) and temporal mechanisms are adaptable in local regions of space (Johnston et al., 2006; Ayhan et al., 2009). The results from this experiment suggest no overwriting of stimuli occurred between presentations in the same region of space, and conversely that there was no better access to representations of stimuli for repeat presentation in the same region of space. This suggests that stimulus duration is not easily retrieved from a visuospatial memory trace. This conclusion is supported to some degree by the finding of Ayhan et al. (2012), who demonstrated that it was not possible to have effective access to the duration of one of a number of elements when post-cued in a visual duration decision task.

Overall, the findings from this study suggest that although there are spatially localized components of the duration encoding mechanism, there is no evidence that temporal information can be retrieved locally from visual short-term memory.

## Ethics Statement

The experiment was approved by the UCL Experimental Psychology Department Ethics Committee. Participants were given an information sheet and encouraged to ask any questions they may have. Written consent was obtained prior to the experiment, and participants were informed that they could end the session at any time, for any reason, and their data would be removed. Only adults took part in the study.

## Author Contributions

CH and NB were involved in the conception, design and interpretation of the paper. AJ and IM supervised the project and were involved in the conception and interpretation of the data. CH was also responsible for acquisition, analysis, and the writing of the manuscript. All authors gave comments on draft versions and final approval of the version submitted.

## Conflict of Interest Statement

The authors declare that the research was conducted in the absence of any commercial or financial relationships that could be construed as a potential conflict of interest.

The reviewer JD and handling Editor declared their shared affiliation, and the handling Editor states that the process nevertheless met the standards of a fair and objective review.

## Footnotes

1 www.crsltd.com/

2 www.mathworks.co.uk/
